# Fatal Pulmonary Tumor Embolic Microangiopathy in Young Lady without Known Primary Malignancy

**DOI:** 10.1155/2014/231081

**Published:** 2014-11-13

**Authors:** Adel Hammodi, M. Ali Al-Azem, Ahmed Hanafy, Talal Nakkar

**Affiliations:** ^1^King Fahad Specialist Hospital, P.O. Box 15215, Dammam 31444, Saudi Arabia; ^2^Critical Care Department, King Fahad Specialist Hospital, P.O. Box 15215, Dammam 31444, Saudi Arabia

## Abstract

Pulmonary embolism (PE) is a common cause of morbidity and mortality in hospitalized patients. Malignancy, prolonged recumbence, and chemotherapy are renowned risk factors for development of clinically significant PE. Cancer exerts a multitude of pathophysiological processes, for example, hypercoagulability and abnormal vessels with sluggish circulation that can lead to PE. One of the peculiar characteristics of tumor cells is their ability to reach the circulation and behave as blood clot—not a metastasis-occluding the pulmonary circulation. We present a case of fatal pulmonary embolism diagnosed histologically to be due to tumor cell embolism.

## 1. Introduction

Hypoxemia and tachypnea are the most serious signs of critical illness. A wide range of disorders can cause such signs with different pathophysiological processes.

The pulmonary artery and its branches are the final destination for any substance greater than 10 microns reaching the venous circulation which could be thrombi, air, amniotic fluid, fat, injected foreign materials, and tumor. It is known that malignancy is a risk factor for thromboembolism and carries a 4-fold risk of thrombosis event than normal population.

Microscopic pulmonary tumor embolism is the presence of multiple aggregates of tumor cell in the small pulmonary arteries, arterioles, and septal capillaries. This syndrome could happen with a number of malignancies including carcinoma of the breast, stomach, pancreas, liver, and prostate.

We present a case of pulmonary tumor embolic microangiopathy (PTEM) caused by occult adenocarcinoma in a lady without any history of malignancy.

## 2. Case Report

A twenty-nine year-old female patient, unemployed, smoker, and married had no children. She was in her usual state of health till 3 weeks prior to presentation, when she started to complain of breathlessness with exertion and progressed over weeks to be on minimal effort interfering with her usual daily activity. She had orthopnea, chest tightness, and pain which was retrosternal, stitching in character, aggravated with coughing, with no specific radiation, and not associated with sweating, nausea, or vomiting. She was evaluated in a private clinic and diagnosed as having pulmonary hypertension and received sildenafil without any subsequent improvement in her condition.

She gave a past history of unintended weight loss of about 13 kg during last 3 months. In addition, she had night fever, night sweats responding to antipyretics.

Four months back, the patient had nausea and vomiting and diagnosed to have* H. pylori* infection, which was treated by standard triple therapy. There was also a history of itchy erythematous skin rash which was managed with topical corticosteroid during that time.

She was investigated for infertility 4 years ago, where she was diagnosed to have polycystic ovary syndrome (PCOS) and fallopian tube obstruction.

There was no previous history of recent travel or immobilization, pulmonary emboli, congenital heart disease, or chronic lung disease. She had a parrot in her house.

There was neither history of contraceptive pill use nor family history of similar attack.

The patient's vital signs revealed blood pressure of 129/73 mmHg, heart rate of 100 beat/minute, respiratory rate of 28 breath/min, oxygen saturation on room air of 87%, and temperature of 36.8°.

She looked distressed, tachypneic, orthopneic, and cyanosed with congested neck veins. Chest examination showed bilaterally diminished air entry with fine basal crepitations up to midlung zones. Auscultation of the heart revealed normal first and second heart sounds with no additional sounds or audible murmur. There was mild epigastric tenderness on abdominal examination. Her extremities showed neither edema, nor clinical evidence of DVT which was confirmed later by venous Doppler study. She was conscious, alert, and oriented to time, place, and person.

Laboratory work-up showed normal liver, renal function tests, and electrolytes as well. CBC revealed anemia with normal leucocytes and eosinophilic counts.

ECG displayed sinus tachycardia. Echocardiography showed severe pulmonary hypertension with estimated pulmonary artery pressure of 78 mmHg, mild to moderate tricuspid regurgitation, septal wall flattening due to right ventricular pressure overload, and normal LV systolic function with ejection fraction of >55%.

Pulmonary function test (PFT) result was moderate restrictive defect with mild reduction of gas diffusion, while the tumor markers results are shown in Table 1.

**Figure 1:**
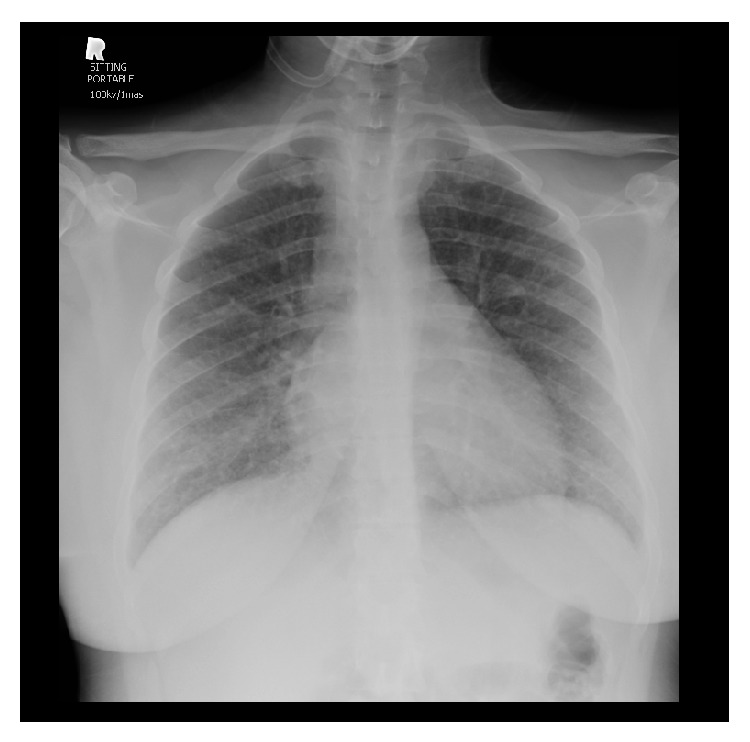


Chest X-ray (Figure 1) revealed bilateral prominent interstitial reticulonodular infiltrate, with no focal pulmonary lesion detected.

**Figure 2:**
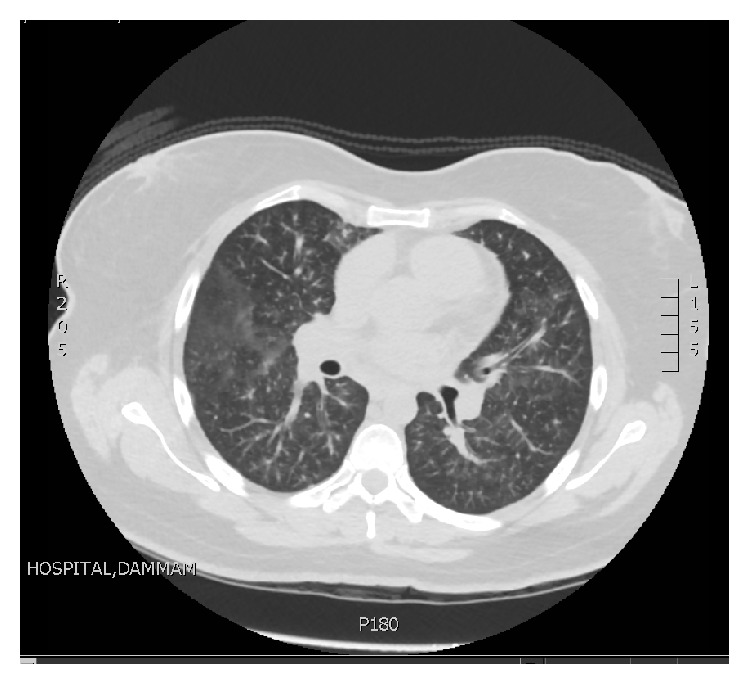


Afterward, high resolution CT of the chest (Figure 2) was done and showed bilateral diffuse ground glass density of the lungs, with centrilobular nodular densities and mild reticulations. However, no volume loss, honeycombing formation, or pleural effusion was seen. Similarly, no significant axillary, hilar, or mediastinal lymph nodes were seen either. Small pericardial effusion was noted. CT Chest PE Study excluded any evidence of pulmonary embolism.

The patient had worsening reticulonodular infiltrations on subsequent chest X-rays, and she became more tachypneic and hypoxic despite the high flow oxygen delivered.

The plan was to obtain an open lung biopsy by thoracic surgeon trying to identify the cause of rapidly worsening interstitial lung disease.

**Figure 3:**
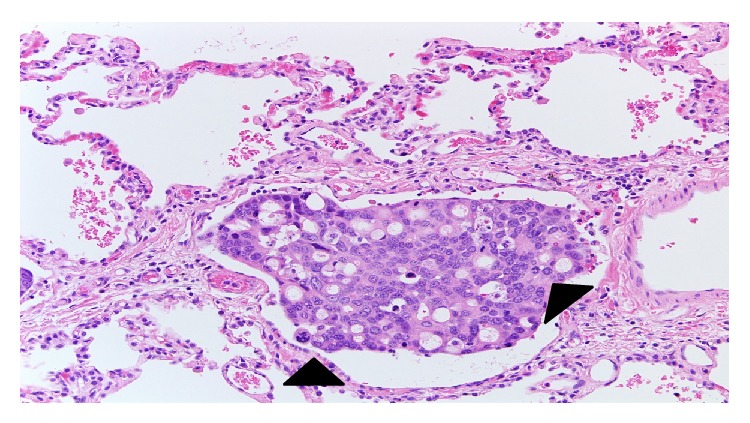


Biopsy result showed (Figure 3) predominantly moderately differentiated adenocarcinoma tumor emboli within vessels (black arrow heads) with no solid tumor mass present.

Immunohistochemical staining was done later trying to identify the primary origin of the tumor and returned as positive for CK7, CK20, CK19, Villin, CEA, and CDx2, focally positive for HER2neu, and negative for ER, PR, GCDFP-15, and TTF-1, supporting a primary GI and pancreaticobiliary origin.

After few days the patient had refractory hypoxemia and developed cardiac arrest, CPR was done but failed, and the patient was declared dead. Autopsy was not done due to family refusal.

## 3. Discussion

The pulmonary tumor thromboembolism syndrome had been portrayed by Kane and Hawkins in 1975 [1].

The incidence of pulmonary tumor thromboembolism depends on the type of malignancy. It is worth noting that, in diagnosed cancer patients, the accurate identification of tumor embolism is made in less than 6% cases [2].

Pulmonary tumor embolism syndrome (also known as pulmonary tumor embolic microangiopathy “PTEM”) has been reported to present 3% to 26% of postmortem examination of cancer patients [3].

The presence of isolated or clusters of tumor cells in the pulmonary arterial circulation is the pathological picture of PTEM [4]. In fact pulmonary tumor emboli (PTEM) are not metastases; they lack the lung specific adhesion molecules and lung specific growth factor.

The incarcerated tumor cells within pulmonary capillaries may activate the coagulation cascade. The occlusion is primarily due to both the tumor cells and the associated clot, but reactive concentric hypertrophy and intimal fibrosis of the pulmonary vessels also contribute [5]. Such histopathological picture is the main reason to term this syndrome as pulmonary tumor embolism microangiopathy (PTEM).

This description is typical to our patient pathology report of the open lung biopsy and explains the restrictive pattern of her PFT.

Not only cancer related factors can affect blood coagulopathy but also a list of known factors including reduced mobility, surgery, chemotherapy, and the use of central venous catheters for chemotherapy and also amplify the risk [6]. Considering our patient's condition, It is obvious that the tumor cells are the main cause of hypoxemia and the embolic event, as she was active and mobilizing few days before her presentation.

Dyspnea, orthopnea, and respiratory failure are frequent complications of malignancy and are often noted near death. Common causes of respiratory failure in patients with cancer are

- pleural effusion,
- infection,
- ARDS,
- lymphangitic carcinomatosis,
- acute cor pulmonale,
- chemotherapy or radiotherapy related restrictive lung disorder,
- thromboembolism,
- primary or secondary involvement mainly endobronchial,
- PTEM.

Nevertheless, survival appears to be poor after the onset of symptoms, probably because pulmonary tumor emboli are often associated with concomitant lymphangitic or metastatic carcinoma [7].

PTEM is rapidly progressing and fatal disorder, as almost all reported cases died within a week from onset, and the same had happened in the current case.

It may be important to diagnose PTEM before development of pulmonary hypertension with early immune-histopathology identification, which may lead to a clinically preferable outcome. Kayatani et al. reported a case of antemortem diagnosis of PTEM which was identified prior to the development of pulmonary hypertension which responded to chemotherapy [8].

Yanagitani et al. also recorded a case of adenocarcinoma of the lung with pulmonary thromboembolism with positive epidermal growth factor receptor (EGFR) which responded well to gefitinib both the embolism and the primary tumor with its metastasis [9].

Our patient was diagnosed after development of severe pulmonary hypertension and her immune-histochemical staining was negative for EGFR, ER, and PR which added to her dismal outcome.

Several ancillary studies may be helpful in distinguishing pulmonary tumor emboli from thromboemboli. For example, ventilation-perfusion lung scans, pulmonary angiography, right heart catheterization and sampling from pulmonary capillaries after wedging, FDG-PET (18F-2-deoxy-2-flouro-D-glucose (FDG) position emission tomography), and lung biopsy were investigated in similar cases [10].

From these investigations, lung biopsy is the gold standard diagnostic test for PTEM.

Another important investigation is echocardiography which differentiates between pulmonary embolism and lymphangitis carcinomatosis [7].

Treatment of PTEM should be directed to the primary tumor. Conventional pulmonary embolism therapies like anticoagulation, thrombolysis, inferior vena, and cava filter placement seem to be ineffective. The main therapeutic options are either surgical resections of the primary tumor, for example, atrial myxoma and renal cell carcinoma, or targeted chemotherapy in case of positive immunohistochemistry [11].

## 4. Conclusion

Pulmonary tumor embolic microangiopathy is a rare and potentially fatal disorder in either diagnosed or undiagnosed cancer patients.

Intensivists should consider tumor embolism along with pulmonary venous thromboembolism as a cause of PE in cancer patients having dyspnea with unremarkable chest X-ray findings.

Early diagnosis may initiate timely appropriate chemotherapy treatment that results in improved outcome of PTEM patients.

## Figures and Tables

**Table 1:** 

	Patient's tumor markers	Normal values upper limit
CA 125	82	0.042 IU/mL
CA 15.3	12	0.027 IU/mL
CA 19.9	13.2	0.037 IU/mL
CEA	89.79	0–5 ug/L
Alpha fetoprotein	1.91	1.09–8.04 ng/mL

## References

[1] Kane R. D., Hawkins H. K., Miller J. A., Noce P. S. (1975). Microscopic pulmonary tumor emboli associated with dyspnea. *Cancer*.

[2] Goldhaber S. Z., Dricker E., Buring J. E., Eberlein K., Godleski J. J., Mayer R. J., Hennekens C. H. (1987). Clinical suspicion of autopsy-proven thrombotic and tumor pulmonary embolism in cancer patients. *The American Heart Journal*.

[3] Keenan N. G., Nicholson A. G., Oldershaw P. J. (2008). Fatal acute pulmonary hypertension caused by pulmonary tumour thrombotic microangiopathy. *International Journal of Cardiology*.

[4] Mehrishi S., Awan A., Mehrishi A., Fein A. (2004). Pulmonary tumor microembolism. *Hospital Physician*.

[5] Goldhaber S. Z., Libby P., Bonow R. O., Mann D. L., Zipes D. P. (2007). Pulmonary embolism. *Braunwald's Heart Disease: A Textbook of Cardiovascular Medicine*.

[6] Fennerty A. (2006). Venous thromboembolic disease and cancer. *Postgraduate Medical Journal*.

[7] Bassiri A. G., Haghighi B., Doyle R. L., Berry G. J., Rizk N. W. (1997). Pulmonary tumor embolism. *The American Journal of Respiratory and Critical Care Medicine*.

[8] Kayatani H., Matsuo K., Ueda Y., Matsushita M., Fujiwara K., Yonei T., Yamadori I., Shigematsu H., Andou A., Sato T. (2012). Pulmonary tumor thrombotic microangiopathy diagnosed antemortem and treated with combination chemotherapy. *Internal Medicine*.

[9] Yanagitani N., Horiike A., Kudo K., Ohyanagi F., Nishio M., Horai T. (2011). A case of adenocarcinoma of the lung with a pulmonary thromboembolism which improved with gefitinib. *Nihon Kokyūki Gakkai Zasshi*.

[10] Masson R. G., Krikorian J., Lukl P., Evans G. L., McGrath J. (1989). Pulmonary microvascular cytology in the diagnosis of lymphangitic carcinomatosis. *The New England Journal of Medicine*.

[11] Seagle R. L., Nomeir A.-M., Watts L. E., Mills S. A., Means W. E. (1985). Left atrial myxoma and atrial septal defect with recurrent pulmonary emboli. *Southern Medical Journal*.

